# Erratum for: Contextual computation by competitive protein dimerization networks

**DOI:** 10.1016/j.cell.2026.03.015

**Published:** 2026-03-18

**Authors:** Jacob Parres-Gold, Matthew Levine, Benjamin Emert, Andrew Stuart, Michael B. Elowitz

In the original version of this article, the citation for Nikitin, 2023 was improperly grouped with two other references that explore competitive dimerization through computational analyses. Nikitin, 2023 demonstrated experimentally that networks of dimerizing DNA strands can perform several input-output computations of interest through a process they term “molecular commutation.” The section of the introduction referring to this work has been updated to more accurately represent its results. The Author Contributions section has also been edited to reflect that three authors, J.P.-G., B.E., and M.B.E., initially conceived the study described in this article, rather than the concept of dimerization network computation in general, and the highlights have been revised to better reflect the conclusions of the study. Additionally, in the web version of the article, the placement of Figure S4 was moved after Figure 5, rather than before. These changes were approved by all co-authors, and no other sections of the article were modified. The authors apologize for any confusion this error may have caused.

