# Supplementary material for: Contextual Computation by Competitive Protein Dimerization Networks
Source: Cell. Author manuscript; Available in PMC 2025 Apr 7. (PMC11973712; doi:10.1016/j.cell.2025.01.036)
Supplement: 3 — Figure S1. An atlas of elementary dimerization network computations, part 1, related to Figure 2 and Figure 4. Shown for each function is a schematic of the network parameters and a plot of the corresponding input-output function, for both one-input functions (top) and a partial set of two-input functions (bottom). The remaining two-input functions can be found in Figure S2. For all panels, the networks shown were inspired by networks from the random parameter screen (Figure 4) and rationally pruned to identify minimal topologies capable of computing each input-output function. All results are displayed in unitless concentrations (see Methods). [file NIHMS2057561-supplement-3.pdf]

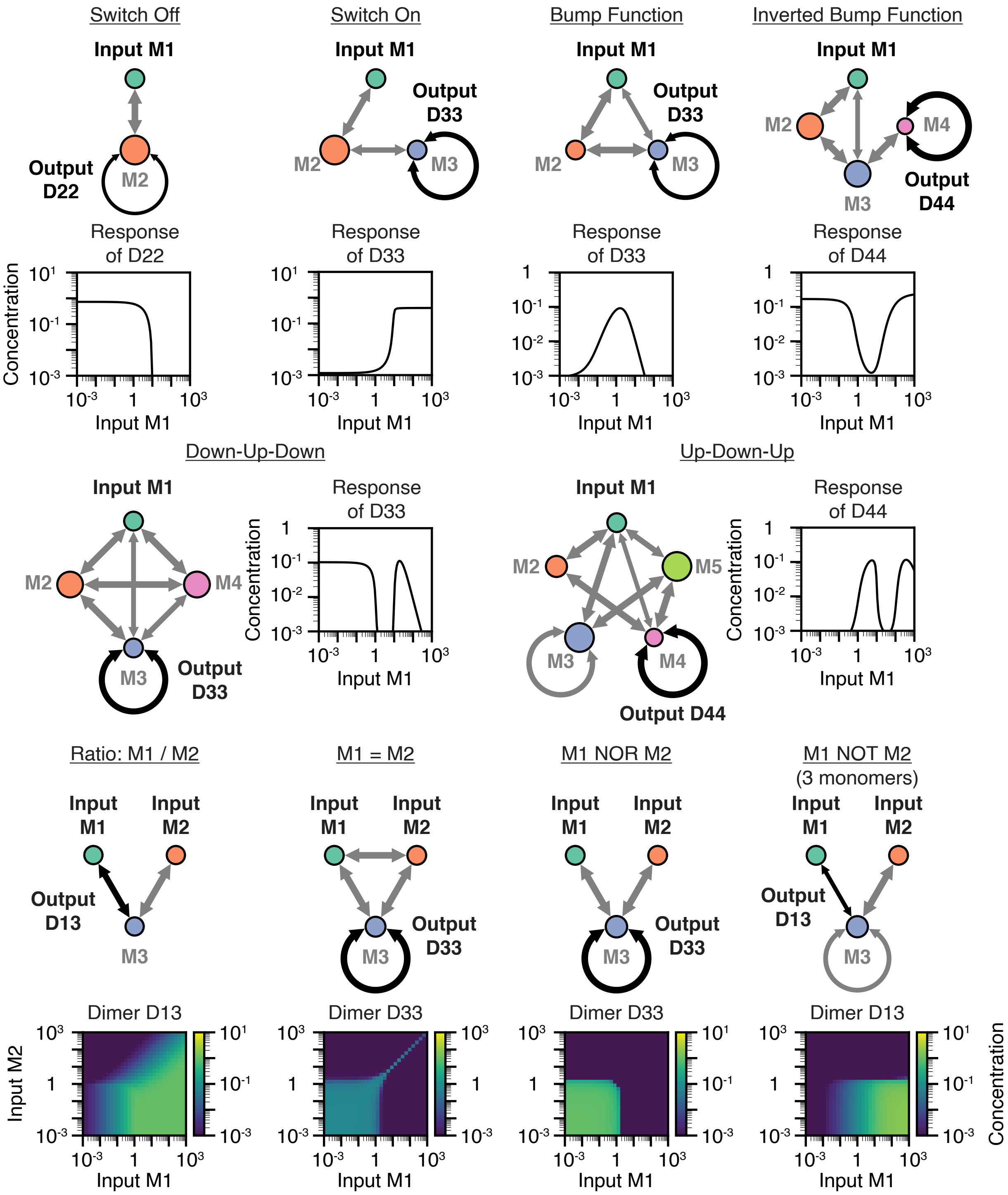

Switch Off
Input M1
Output D22
M2
Response of D22
Concentration
10^{1}
10^{-1}
10^{-3}
Input M1
Switch On
Input M1
Output D33
M2
M3
Response of D33
Bump Function
Input M1
Output D33
M2
M3
Response of D33
Inverted Bump Function
Input M1
M2
M4
M3
Output D44
Response of D44
1
10^{-1}
10^{-2}
10^{-3}
10^{3}
Down-Up-Down
Input M1
M2
M4
M3
Output D33
Response of D33
Up-Down-Up
Input M1
M2
M5
M3
M4
Output D44
Response of D44
Ratio: M1 / M2
Input M1
Input M2
Output D13
M3
M1 = M2
Output D33
M1 NOR M2
M1 NOT M2 (3 monomers)
Dimer D13
Dimer D33
Input M2
Input M1
Concentration
