# Supplementary figures and images for: Contextual Computation by Competitive Protein Dimerization Networks
Source: Cell. Author manuscript; Available in PMC 2025 Apr 7. (PMC11973712; doi:10.1016/j.cell.2025.01.036)

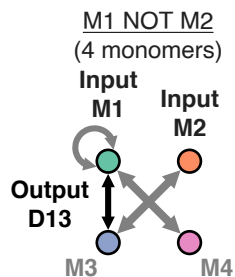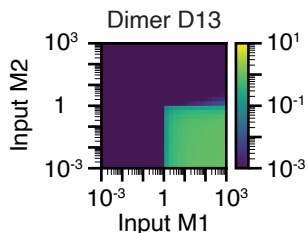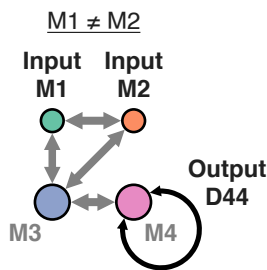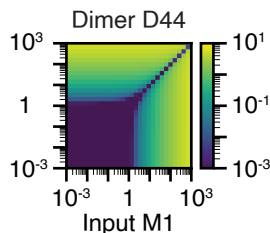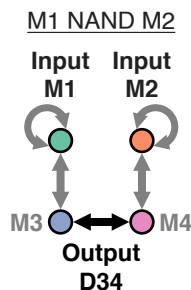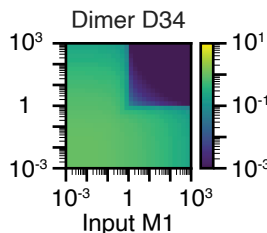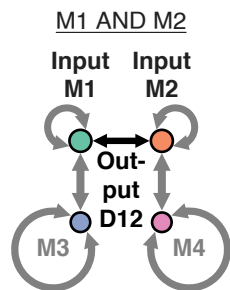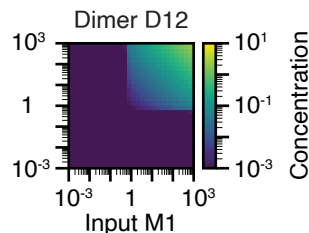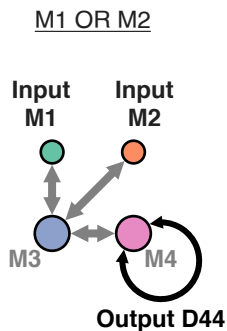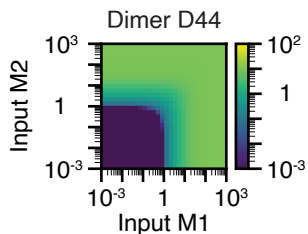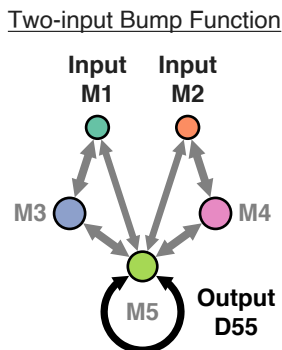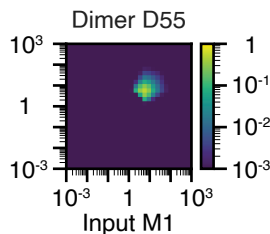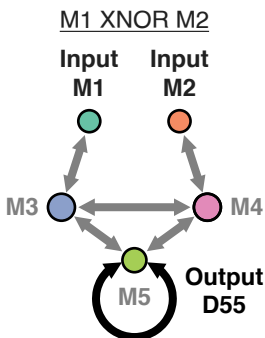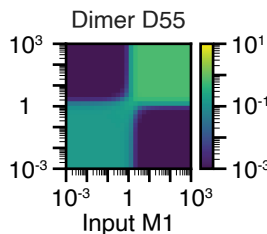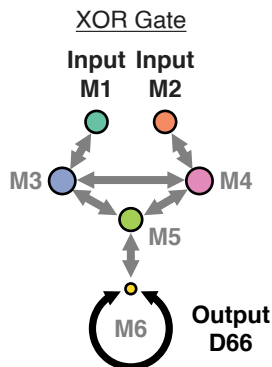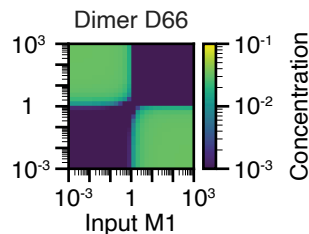

Supplement: 4 — Figure S2. An atlas of elementary network computations, part 2, related to Figure 2 and Figure 4. Shown for each function is a schematic of the network parameters and a plot of the corresponding input-output function. Displayed are the two-input computations not included in Figure S1. For all panels, the networks shown were inspired by networks from the random parameter screen (Figure 4) and rationally pruned to identify minimal topologies capable of computing each input-output function. All results are displayed in unitless concentrations (see Methods). [file NIHMS2057561-supplement-4.pdf]

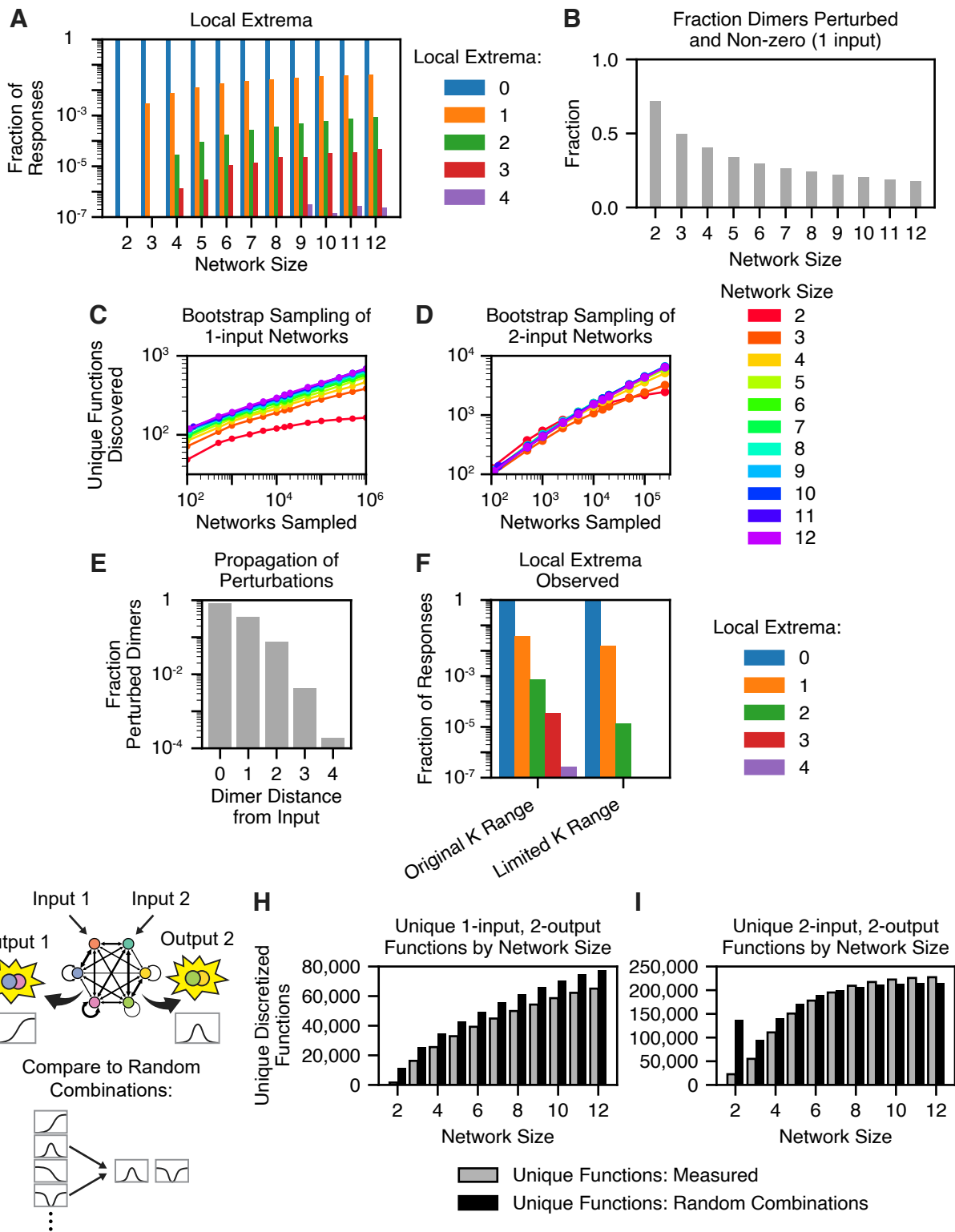

Supplement: 5 — Figure S3. A global parameter screen reveals the diversity and nature of dimerization network computations, related to Figure 4. (A) A bar graph shows, for each network size, the fraction of responses with zero to four local extrema (i.e., local minima and maxima). (B) A bar graph shows, for each network size, the fraction of dimers that both form at significant concentrations and are perturbed more than 10-fold by a titration of the input monomer. (C-D) The number of unique one-input (C) and two-input (D) functions observed is plotted versus the number of networks sampled in the random parameter screen. (E) A bar graph shows, for increasing distances between the input monomer and output dimer, the fraction of dimers (out of all dimers that form at appreciable concentrations) that are perturbed more than 10-fold in response to a titration of the input monomer. (F) A bar graph shows, for a parameter screen of 12-monomer networks using a more limited range of affinities Kij from 10−3) to 101, the fraction of responses with zero to four local extrema (i.e., local minima and maxima). (G) A schematic depicting how two dimers within the same network could be used to compute two-output functions. (H) A bar graph shows, for each network size, the number of unique, discretized, one-input, two-output functions, as well as the number of unique functions for a scrambled control in which random pairs of response functions were selected from the overall dataset. (I) A bar graph shows, for each network size, the number of unique, discretized, two-input, two-output functions, as well as the number of unique functions for a scrambled control in which random pairs of response functions were selected from the overall dataset. The outlier for the m=2 scrambled data appears to be due to the m=2 dataset having a more even distribution of unique functions among the whole set of responses. [file NIHMS2057561-supplement-5.pdf]

**A**

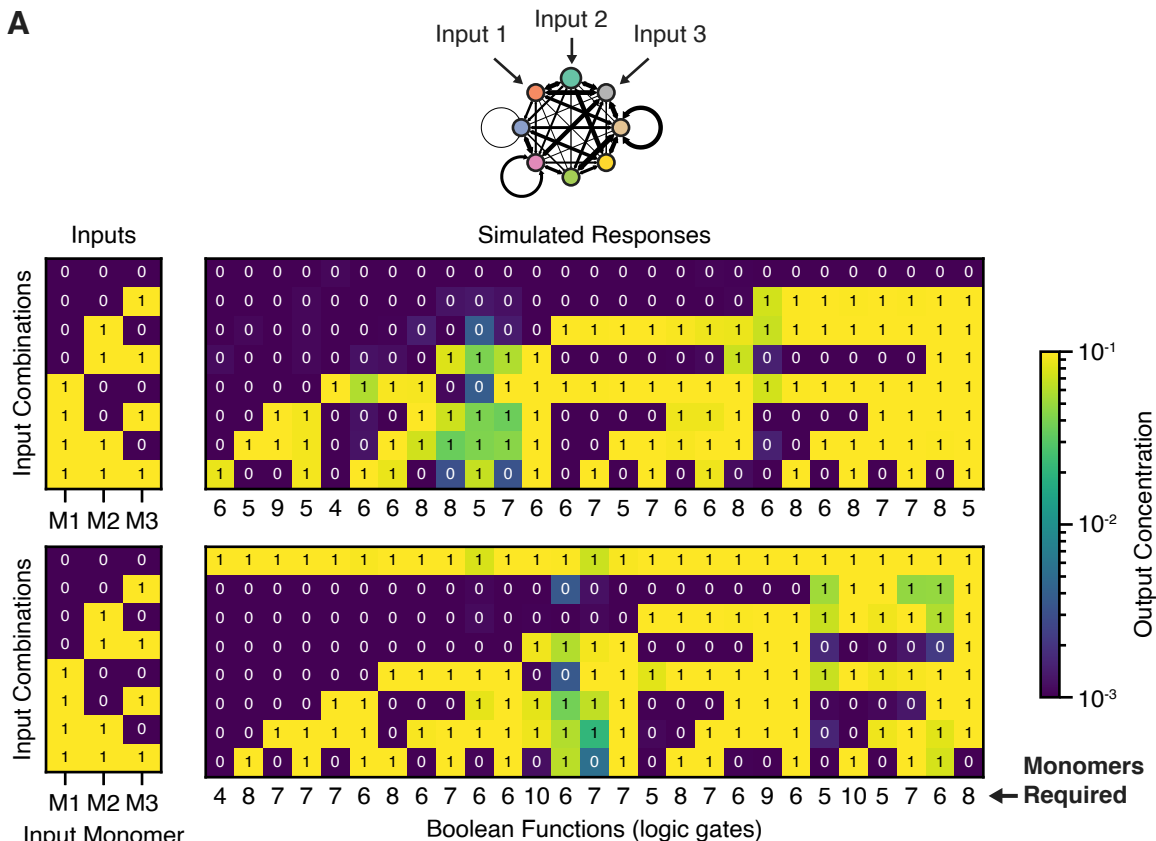

**B**

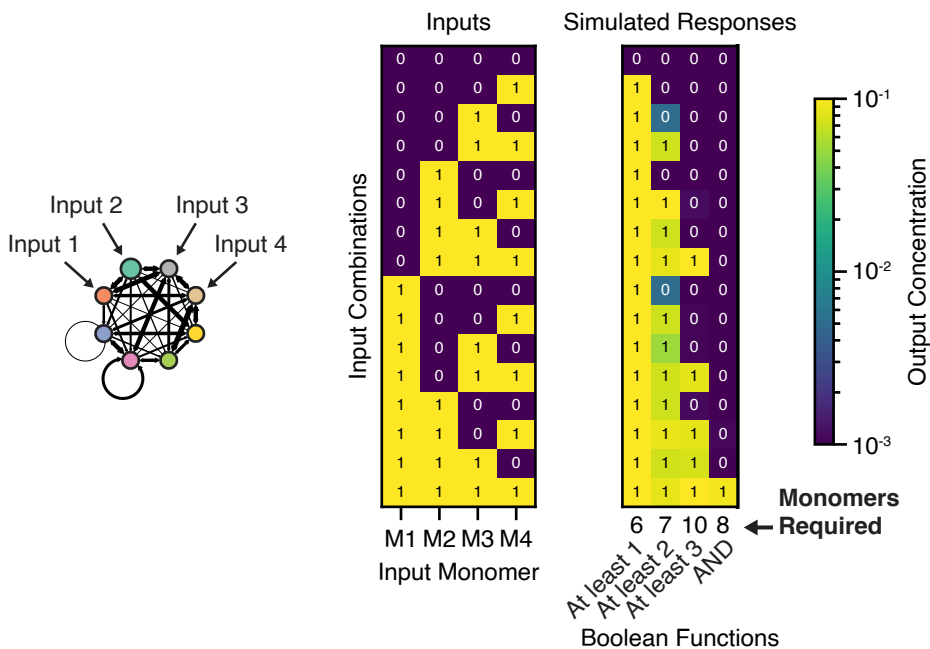

Supplement: 6 — Figure S4. Competitive dimerization networks can compute multi-input functions, related to Figure 5. (A) Dimerization networks can perform all three-input logic gates. (left) Rows represent different combinations of inputs that are presented to each network. (right) A heatmap of responses is shown, where each column represents a unique logic gate and the color of the response heatmap represents the output dimer concentration. The number of network monomers required to perform each gate is noted below each column. (B) Dimerization networks can perform four-input logic gates. Shown are four examples, the AND and “at least n” gates, which output 1 if at least n inputs are present. [file NIHMS2057561-supplement-6.pdf]

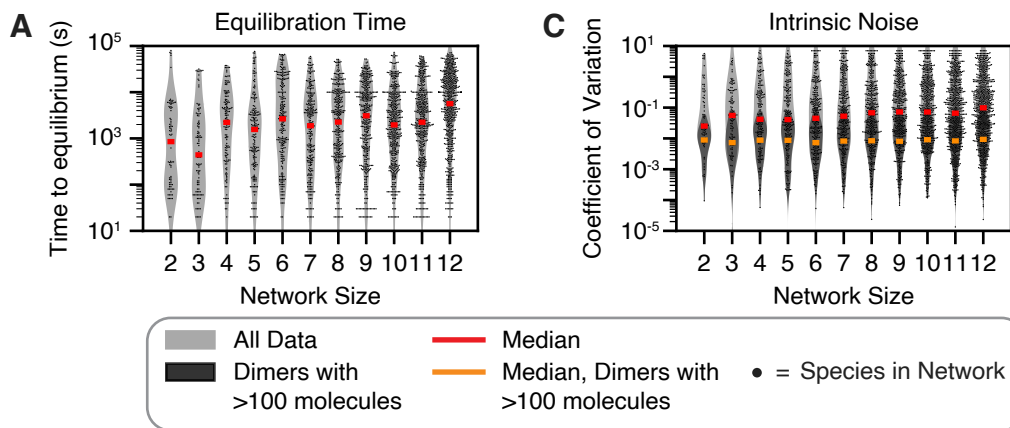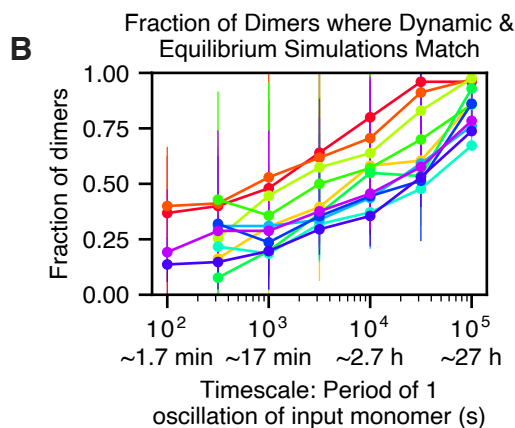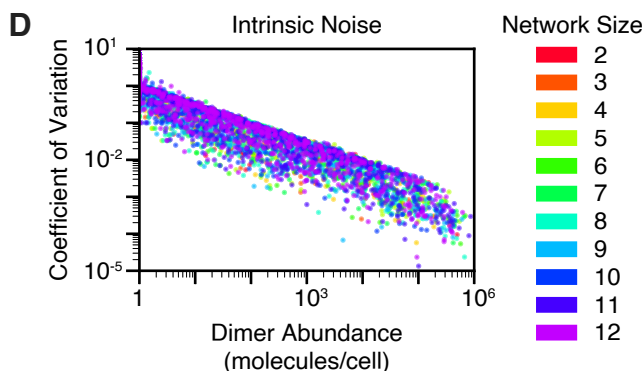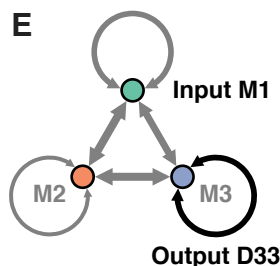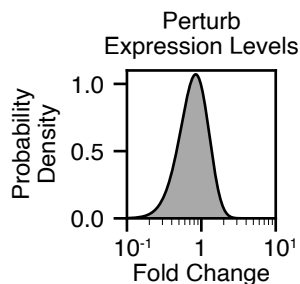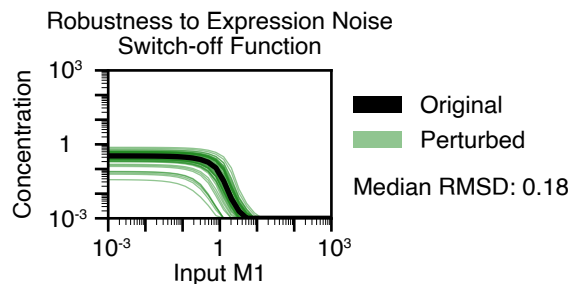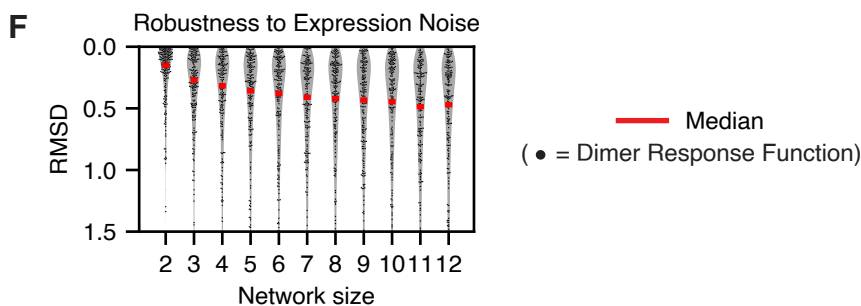

Supplement: 7 — Figure S5. Competitive dimerization networks exhibit biologically reasonable equilibration kinetics and robustness to noise, related to Figure 4. (A) Network equilibration kinetics were simulated by numerically integrating ordinary differential equations (ODEs) describing dimer association and dissociation kinetics. The time for each species in n=20 networks to re-equilibrate after a perturbation of an input monomer is displayed as a violin plot with scattered points. (B) To assess the timescale at which a dynamical dimerization network can no longer be assumed to be at equilibrium, we simulated network equilibration as the total concentration of one monomer was oscillated sinusoidally. For each dimer, we compared the dynamical trajectory of its concentration to the calculated equilibrium concentration of the dimer at that timepoint. Shown is the fraction of dimers (out of all dimers whose concentrations change significantly over the course of the simulation) for which the dynamical and equilibrium concentrations matched at every timepoint (within 0.5 log units, or less than ~3-fold difference), for various network sizes m as well as different timescales at which one monomer was perturbed sinusoidally. n=10 different networks of each network size were simulated; the error bars show the 1st and 3rd quartiles of the data across different networks. Points for the 100 s timescale with network sizes 4–10 were not shown, as 40–70% of these simulations failed numerically. (C-D) The intrinsic noise of the binding equilibrium was simulated using the Gillespie algorithm with 100 steps of 10 s each. (C) A violin plot (light gray) with scattered points shows the coefficient of variation, a measure of noise, for each species. A dark gray violin shows the data specifically for species present at high abundances (>100 molecules/cell, median shown by the orange line). (D) A scatterplot shows the relationship between the equilibrium abundance (in molecules/cell) and the intrinsic no [file NIHMS2057561-supplement-7.pdf]
