# Supplementary material for: Contextual Computation by Competitive Protein Dimerization Networks
Source: Cell. Author manuscript; Available in PMC 2025 Apr 7. (PMC11973712; doi:10.1016/j.cell.2025.01.036)
Supplement: 8 — Figure S6. The versatility of random networks increases with network size, related to Figure 6. (A) A scatter plot shows how network versatility using only a single output dimer (left) or any output dimer (right) varies with network connectivity. (B) To show how difficult each target function was to achieve, a violin plot with scattered points shows the fraction of random networks that could achieve each target function, separated by network size and whether only a single dimer (left) or any dimer (right) may be used as the output. (C) While Figure 6B shows only the fraction of targets that could be achieved by each network, plotted here is the projected total number of targets each network is expected to achieve – accounting for differences in the overall expressivities of networks differing in size – for both cases in which only a single dimer (top) or any dimer (bottom) may be used as the output. (D) A scatterplot showing, for all combinations of target functions achieved in the versatility analysis by networks with m=8 monomers, both the maximum log fold change in accessory expression level (maximum over all accessory monomers) and the Euclidean distance between the two target functions. (E) A violin plot with scattered points shows the versatility of n=10 random networks with m=20 monomers toward t=10 2-input target functions at different thresholds of the Pearson correlation coefficient. (F) An array showing the responses of all n=10 random networks optimized to perform t=10 named 2-input target functions. For the violin plots, the gray violins show the kernel density estimate of the data distributions, red lines show the median values, and only a random subset of the data is displayed as scattered points. [file NIHMS2057561-supplement-8.pdf]

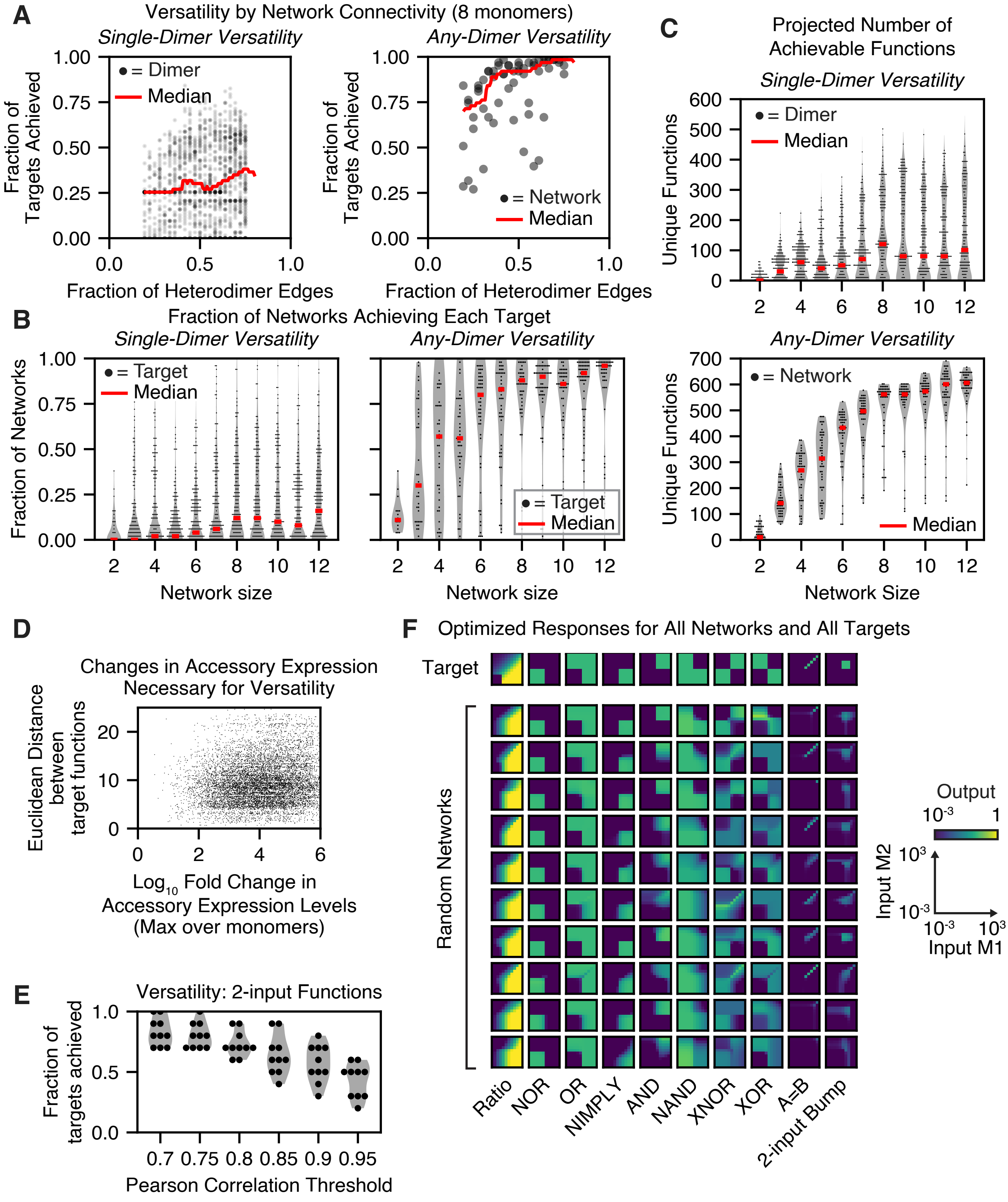
A
Versatility by Network Connectivity (8 monomers)
Single-Dimer Versatility
Any-Dimer Versatility
Fraction of Targets Achieved
Fraction of Heterodimer Edges
= Dimer
Median
= Network
B
Fraction of Networks Achieving Each Target
Fraction of Networks
Network size
= Target
C
Projected Number of Achievable Functions
Unique Functions
Network Size
D
Changes in Accessory Expression Necessary for Versatility
Euclidean Distance between target functions
Log10 Fold Change in Accessory Expression Levels (Max over monomers)
E
Versatility: 2-input Functions
Fraction of targets achieved
Pearson Correlation Threshold
F
Optimized Responses for All Networks and All Targets
Target
Random Networks
Ratio
NOR
OR
NIMPLY
AND
NAND
XNOR
XOR
A=B
2-input Bump
Output
Input M2
Input M1
