# Supplementary material for: Contextual Computation by Competitive Protein Dimerization Networks
Source: Cell. Author manuscript; Available in PMC 2025 Apr 7. (PMC11973712; doi:10.1016/j.cell.2025.01.036)
Supplement: 9 — Figure S7. Natural dimerization networks are of sufficient size to exhibit high expressivity and versatility, related to Figure 4 and Figure 6. (A, B) Co-expression of bZIP (A) and nuclear receptor (NR) (B) transcription factors was assessed for many cell types across both mouse and human datasets. A violin plot with scattered points shows the number of network proteins co-expressed in each cell type. Gray violins show the kernel density estimate of the data distributions, red lines show the median values, and black dotted lines indicate the total number of genes assessed. (C) Table summarizing the size, number of known interacting members, number of co-expressed members, and connectivity of several natural dimerization networks. N.D. indicates that an entry was not determined. [file NIHMS2057561-supplement-9.pdf]

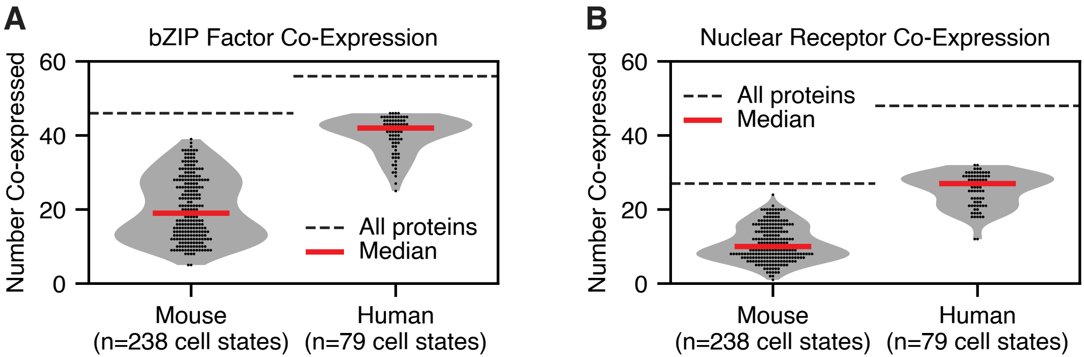

C

Organism, Protein Family	Family Members	Interacting Members	Co-expressed (median)	Connectivity	Refs
Human (*homo sapiens*) bZIP	57	> 21	42	20%	Reinke et al. 2013
Mouse (*mus musculus*) bZIP	46	N. D.	15	N. D.	
Arabidopsis thaliana bZIP	78	> 14	N. D.	24%	Llorca et al. 2015
Human (*homo sapiens*) Nuclear Receptor	48	> 30	28	28%	Amoutzias et al. 2007
Mouse (*mus musculus*) Nuclear Receptor	27	N. D.	7	N. D.	
Arabidopsis thaliana MADS-box	107	> 11	N. D.	40%	de Folter et al. 2005
